# What’s past is prologue: epigenetic memory links transient inflammation to future disease

**DOI:** 10.1038/s41392-026-02877-0

**Published:** 2026-07-01

**Authors:** Samuel T. Keating, Assam El-Osta

**Affiliations:** 1 Resonate, Communication Consultancy, Copenhagen, Denmark; 2 Baker Heart and Diabetes Institute, Epigenetics in Human Health and Disease Program, Melbourne, VIC Australia; 3 Baker Department of Cardiometabolic Health, The University of Melbourne, Parkville, VIC Australia; 4 School of Translational Medicine, Department of Diabetes, Monash University, Melbourne, VIC Australia; 5 Department of Medicine and Therapeutics, The Chinese University of Hong Kong (CUHK), Hong Kong SAR, China

**Keywords:** Epigenetic memory, Experimental models of disease

In recent studies published in *Science* and *Nature*, Cowley et al.^1^ and Nagaraja et al.^2^ show that transient inflammatory injury can be converted into durable epigenetic memory through selective chromatin persistence, incomplete erasure after recovery, and lineage propagation with later functional consequences, including tumour-promoting susceptibility. Together, they expose the fiction that recovery restores true biological innocence, shifting the discussion from the vague language of cellular memory to a more exacting mechanistic account of persistence, inheritance, and future disease risk.

The conceptual stakes are considerable. A field long preoccupied with inducible transcriptional responses has struggled to explain why some stress-evoked chromatin states resolve with recovery whereas others endure long after the initiating stimulus has disappeared. Earlier work on hyperglycaemic memory established the principle that brief metabolic injury can leave lasting molecular residue, showing that transient hyperglycaemia sustained endothelial activation during subsequent normoglycaemia through persistent expression of the NF-κB subunit p65 and enduring chromatin remodelling at its promoter.^3^ That work was important because it made the unsettling point that exposure can outlast itself. What it did not fully explain was why only certain loci, in certain cellular contexts, acquire true epigenetic longevity. The new studies advance that question with greater scale, sharper mechanistic resolution and, crucially, more direct relevance to later disease.

Cowley et al. address the most difficult aspect of the problem: durability. Using epidermal stem cells exposed to transient psoriasis-like inflammation, they show that most inflammatory memory domains erode over time, but a restricted subset persists across striking timescales, with functional consequences detectable throughout the murine lifespan. Their central claim is both elegant and consequential: longevity is not adequately explained by residual inflammatory transcription factors or enhancer-associated histone marks alone. Instead, long-term memory domains are distinguished by an underlying regulatory architecture, particularly CpG enrichment, that predisposes them to persistence once activated. These loci undergo durable demethylation, favour binding by methylation-sensitive factors, display intrinsic nucleosome-disfavouring properties, and acquire the histone variant H2A.Z, thereby stabilising accessibility through time and cell division. This is the study’s real conceptual achievement. It argues that inflammatory transcription factors may open the door, but DNA sequence helps decide which regulatory regions remain epigenetically ajar.

That insight is more important than the technical sophistication alone. The paper’s value lies not simply in cataloguing persistent domains with impressive multi-omic resolution, but in offering a plausible grammar for epigenetic longevity. It is a stronger study conceptually than therapeutically: it clarifies why persistence of chromatin state is selective, but it does not yet show how that selectivity might be manipulated with precision. Even so, it gives the field something firmer than metaphor: a mechanistic account of persistence. Long-term inflammatory memory is no longer treated as a vague after-effect of prior insult, but as a molecularly encoded feature of regulatory elements.

Nagaraja et al. ask the next and more unsettling question: what does remembered injury do to a tissue? In a mouse model of recurrent colitis, they show that colonic stem cells retain an epigenetic memory of inflammation for more than 100 days after apparent disease resolution. Transcriptional profiles and tissue morphology largely return to baseline, but chromatin accessibility does not. The remembered state is marked by cumulative gain in AP-1 motif accessibility, persists cell-intrinsically, and is clonally inherited through stem-cell divisions, with some lineages retaining far stronger memory than others. Most importantly, this remembered state is not benign. Upon oncogenic challenge, previously inflamed tissue generates larger tumours, indicating that prior inflammatory experience lowers the threshold for malignant outgrowth. The paper’s force lies here: it makes the historical record of inflammation legible not merely as molecular persistence, but as a de facto memory that manifests as future biological vulnerability. Nor is this logic confined to barrier tissues. Recent work in the central nervous system has identified a disease-associated astrocyte memory state in which repeated inflammatory stimulation elicits stronger pro-inflammatory recall responses through ACLY-p300-dependent histone acetylation and increased chromatin accessibility, extending the concept of inflammatory memory to pathogenic glial states relevant to experimental autoimmune encephalomyelitis and multiple sclerosis.^4^

This is a biologically compelling study, though its claims should be read with discipline. It shows that inflammatory experience can leave a clonally heritable chromatin scar that later amplifies tumorigenesis in an experimentally tractable setting. What it does not yet establish is whether such memory is a universal driver of malignancy, or a context-dependent permissive state whose pathological effect depends on subsequent oncogenic insult, tissue ecology, and lineage history. That distinction matters. The work is translationally suggestive, but not yet decisive. Still, it sharpens the field’s thinking by placing epigenetic memory in an active mechanistic circuit rather than an archival one: prior injury does not simply leave a temporal imprint; it rewrites the terms of future response.

Read together, the two papers are complementary in the strongest sense. The *Science* study explains why only a subset of stress-responsive loci acquires genuine epigenetic longevity. The *Nature* study shows why that persistence matters. One dissects the logic of cellular memory, the other exposes its pathology. Their combined contribution is to recast epigenetic memory as selective, hierarchical, and biologically consequential. Most stress-induced chromatin changes resolve. Only some are licensed for persistence. And only a fraction of those may later prove dangerous. That hierarchy helps resolve a long-standing tension in the field: if histone modifications and inducible transcription factors are often labile, how can memory survive tissue turnover and repeated cell division? Cowley et al. offer one answer with unusual clarity: long-term memory is not merely imposed upon chromatin by transient upstream factors but stabilised from within by the sequence logic of particular regulatory domains. Nagaraja et al. then show that such stabilised states can acquire pathological force in a regenerative tissue under oncogenic pressure.

The broader implication is now difficult to ignore. Recovery may restore appearance without erasing the underlying molecular memory. A tissue can look healed while retaining a molecular scar that alters its next response to injury, inflammation, or transformation. Sometimes such memory may be adaptive, enabling faster repair, heightened resilience, or enhanced responsiveness. But the new work makes clear that remembered inflammation also exacts a price. In the colon, it may create a permissive substrate for tumour growth. In other contexts, by extension, it may sustain chronic susceptibility long after the initiating insult has vanished. The old promoter-centric model of stress memory, though still valuable, now looks too narrow. Durable memory is better understood as a systems property of selected regulatory domains, stem-cell lineages and chromatin features that together determine whether experience fades or hardens into biological precedent (Fig. 1).

**Fig. 1:**
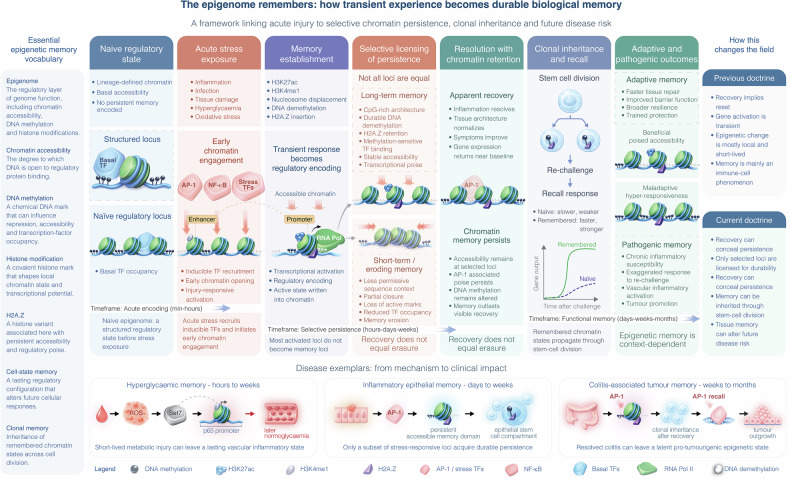
The epigenome remembers: how transient experience becomes durable biological memory. This schematic outlines a framework by which transient injury or inflammatory exposure can be converted into durable biological memory through selective chromatin persistence, incomplete erasure during recovery, and inheritance through stem-cell division. In the naïve regulatory state, chromatin is lineage-defined and basally accessible, with no persistent memory encoded. Acute stress exposure, including inflammation, infection, tissue damage, hyperglycaemia and oxidative stress, recruits inducible transcription factors such as AP-1 and NF-κB, initiating early chromatin opening and injury-responsive activation. At a subset of loci, this transient response progresses to memory establishment, marked by increased accessibility, enhancer-promoter communication, RNA polymerase II recruitment, nucleosome displacement, reduced DNA methylation, and acquisition of activating chromatin features, including H3K27ac, H3K4me1 and H2A.Z. The model then distinguishes loci that undergo selective licensing of persistence from those that erode. Long-term memory loci are associated with CpG-rich architecture, durable DNA demethylation, H2A.Z retention, methylation-sensitive transcription-factor binding, stable accessibility and transcriptional poise, whereas short-term loci progressively lose active features and accessibility. During resolution with chromatin retention, tissue architecture and apparent phenotype recover, but accessibility, altered methylation states and AP-1-associated poise can persist at selected regulatory regions, such that recovery does not necessarily equal erasure. These remembered states may then undergo clonal inheritance and recall, propagating through stem-cell division and enabling faster, stronger responses upon re-challenge than in naïve cells. The framework further distinguishes adaptive from pathogenic outcomes. In some contexts, retained memory may support faster repair, improved barrier function and resilience; in others, it may predispose to chronic inflammatory susceptibility, exaggerated re-challenge responses, vascular inflammatory activation and tumour promotion. The figure therefore contrasts a previous doctrine, in which recovery implies reset and epigenetic change is transient, with a current doctrine in which only selected loci are licensed for durability and tissue memory can alter future disease risk. Disease examples illustrate hyperglycaemic memory, inflammatory epithelial memory and colitis-associated tumour memory, emphasizing that resolved injury can leave latent, biologically consequential epigenetic states. Symbols denote DNA methylation, DNA demethylation, H3K27ac, H3K4me1, H2A.Z, basal and inducible transcription factors, and RNA polymerase II. Figure was prepared using Adobe Illustrator (v30.5.1) and Adobe Photoshop (v27.7)

The principal limitation is equally clear: neither study shows how to selectively erase maladaptive memory while preserving the adaptive competence that enables tissues to respond to future stress. Yet recent work in the central nervous system suggests that this question may be mechanistically tractable. In astrocytes, early loss of the glucocorticoid receptor NR3C1 induces long-lasting epigenetic priming of inflammatory genes, increasing later susceptibility to exaggerated immune activation, whereas intact NR3C1-mediated regulation suppresses these responses through context-dependent interactions involving AP-1 and NF-κB.^5^ That question now becomes unavoidable. If prior injury can become molecular precedent, then the next frontier is not simply to map remembered states, but to distinguish the useful from the dangerous and to intervene before persistence becomes pathology. The question is no longer whether cells remember. It is whether biology can learn to forget. Recovery may heal, but history endures.

## References

[1] Cowley, C. J. et al. Distinctive DNA sequence features define epigenetic longevity of inflammatory memory. *Science* **391**, eadz6830 (2026). 41886579 10.1126/science.adz6830 PMC13295011

[2] Nagaraja, S. et al. Epigenetic memory of colitis promotes tumour growth. *Nature* **652**, 774–783 (2026). 41882356 10.1038/s41586-026-10258-4 PMC13083248

[3] El-Osta, A. et al. Transient high glucose causes persistent epigenetic changes and altered gene expression during subsequent normoglycemia. *J. Exp. Med.* **205**, 2409–2417 (2008). 18809715 10.1084/jem.20081188 PMC2556800

[4] Lee, H. G. et al. Disease-associated astrocyte epigenetic memory promotes CNS pathology. *Nature* **627**, 865–872 (2024). 38509377 10.1038/s41586-024-07187-5 PMC11016191

[5] Park, S. et al. NR3C1-mediated epigenetic regulation suppresses astrocytic immune responses in mice. *Nat. Commun.* **16**, 8330 (2025). 40983615 10.1038/s41467-025-64088-5 PMC12454645

